# Pulmonary Complications after Surgery for Rectal Cancer in Elderly Patients: Evaluation of Laparoscopic versus Open Approach from a Multicenter Study on 477 Consecutive Cases

**DOI:** 10.1155/2017/5893890

**Published:** 2017-10-22

**Authors:** Marco Milone, Ugo Elmore, Andrea Vignali, Alfredo Mellano, Nicola Gennarelli, Michele Manigrasso, Francesco Milone, Giovanni Domenico De Palma, Andrea Muratore, Riccardo Rosati

**Affiliations:** ^1^Department of Surgical Specialities and Nephrology, University of Naples “Federico II”, Naples, Italy; ^2^Department of Gastrointestinal Surgery, San Raffaele Hospital, Milan, Italy; ^3^Department of Surgical Oncology, Candiolo Cancer Institute-FPO IRCCS, Candiolo, Turin, Italy

## Abstract

**Aim:**

To evaluate the impact of open or laparoscopic rectal surgery on pulmonary complications in elderly (>75 years old) patients.

**Methods:**

Data from consecutive patients who underwent elective laparoscopic or open rectal surgery for cancer were collected prospectively from 3 institutions. Pulmonary complications were defined according to the ACS/NSQUIP definition.

**Results:**

A total of 477 patients (laparoscopic group: 242, open group: 235) were included in the analysis. Postoperative pulmonary complications were significantly more common after open surgery (8 out of 242 patients (3.3%) versus 23 out of 235 patients (9.8%); *p* = 0.005). In addition, PPC occurrence was associated with the increasing of postoperative pain (5.04 ± 1.62 versus 5.03 ± 1.58; *p* = 0.001) and the increasing of operative time (270.06 ± 51.49 versus 237.37 ± 65.97; *p* = 0.001).

**Conclusion:**

Our results are encouraging to consider laparoscopic surgery a safety and effective way to treat rectal cancer in elderly patients, highlighting that laparoscopic surgery reduces the occurrence of postoperative pulmonary complications.

## 1. Introduction

With the global rise in life expectancy and the increasing incidence of colorectal cancer, more and more elderly patients are diagnosed with this disease [1]. The appropriate approach to surgical intervention in these high-risk patients is extremely actual. Recent population-based data suggest that this vulnerable population can benefit from the use of less invasive surgical approaches [2, 3]. However, concern has been raised over the safety of laparoscopic surgery for older patients, given their lower physiological reserves, and the necessity to establish and to maintain pneumoperitoneum which is added to the physiological stress experienced by the patients during laparoscopic surgery. This is particularly true for individuals with suboptimal pulmonary function with subsequent risk of cardio and pulmonary complications [3, 4]. This is of relevance in elderly patients who are candidates for laparoscopic rectal resection which requires longer head-down tilt position (Trendelenburg position). Data from recent meta-analysis, however, report lower cardiopulmonary complications in elderly patients who undergone laparoscopic colorectal resection suggesting that mini-invasive approach should be aggressively applied to these high-risk patients [5]. Nevertheless, high-quality evidence regarding the benefits of laparoscopic rectal surgery in this patient group is limited, as these patients are frequently excluded from randomized trials [6–8], and right now, there is insufficient evidence to draw conclusions on the effect of laparoscopy on the incidence and severity of pulmonary complications in elderly patients undergoing laparoscopic rectal resection [5]. Moreover, the benefit of laparoscopic surgery for rectal cancer is still controversial [9–12].

The aim of this study was to evaluate the influence of open or laparoscopic surgery on pulmonary complications in elderly patients with resettable rectal cancer.

## 2. Methods

A chart review has been performed on elderly patients of both sexes (age: 75 years old or more) who underwent elective rectal surgery, from January 2009 to December 2015, at the Department of Surgery of IRCCS Humanitas Research Hospital, at the Department of Surgical Oncology in Candiolo (Turin) IRCCS Cancer Institute, and at the Department of Surgical Specialities of the University of Naples “Federico II”. Institutional review board approval was obtained before the review of any patient material. Written informed consent was obtained from all study participants or their legal guardian before the enrolment in the study.

Inclusion criteria were as follows: age 75 years old or more, histologically documented cancer of the rectum, surgical operation performed by expert surgeons (more than 50 laparoscopic rectal resections performed), standardized surgical technique, and standardized perioperative management. The localization of the tumour in the rectum was categorized as upper (distal border of the tumour 10–15 cm from the anal verge), middle (5–10 cm), and lower rectum (<5 cm).

Exclusion criteria were emergency surgery, cancer infiltrating adjacent organ/s, and contraindication to a laparoscopic procedure. Therefore, a case-controlled study has been designed, including 242 patients who underwent laparoscopic rectal resection compared with 235 patients who underwent open rectal resection during the same period.

Open (OS) or laparoscopic surgery (LS) has been performed according to the clinical advice of each individual surgeon. A propensity score analysis has been performed to exclude any bias related to the allocation of each patient in the different study groups.

### 2.1. Surgical Details

The procedures performed by an expert surgeon (more than 50 laparoscopic rectal resection) have been included in the study. All procedures had to comply with the principles of total mesorectal excision (TME) or partial mesorectal excision (PME) using our previously published technique [13]. TME requires the removal of the entire mesorectum down to the pelvic floor. It was done both with preservation of the anal sphincter and with abdominoperineal resection. Rectal cancers located in the upper part of the rectum can be resected with sufficient margin by transecting the mesorectum at about 5 cm distally from the lower margin of the tumour, resulting in PME. Completion of laparoscopic dissection of the mesorectum was judged as necessary to qualify a procedure as laparoscopic. If the mesorectal dissection has been completed by an open approach, the procedure was judged as conversion to open surgery.

### 2.2. Postoperative Care

All patients were treated according to a standardized ERAS perioperative care protocol as explained in our previously published experience [13]. In details, the nasogastric tube was removed immediately after surgery in all patients.

Short-term follow-up was conducted at 15 and 30 days after discharge. All adverse events that occurred within 30 days after surgery were considered complications. Patients with early surgical complications (leak, bleeding, or any infection) were excluded from analysis to exclude the influence of the complication on the pneumonia occurrence.

### 2.3. Outcomes

The primary endpoint is the proportion of pulmonary complications after surgery. The term “pulmonary complications” defines all conditions according to the American College of Surgeons National Surgical Quality Improvement Program's (ACS NSQIP's) definition which includes postoperative pneumonia, prolonged mechanical ventilation beyond 48 hours, and unplanned intubation within 30 days of surgery. Secondary outcomes to be evaluated were age, gender, body habitus, comorbidities, ASA score, tumour localization, tumour stage, operative time, and pain score at 24 and 48 hours after surgery.

### 2.4. Statistical Analysis

Statistical analysis was performed with the SPSS 16 system (SPSS Inc., Chicago, IL, USA). Continuous data were expressed as mean ± SD; categorical variables were expressed as %. To compare continuous variables, an independent sample *t*-test was performed. The Wilcoxon test for paired samples was employed as a nonparametric equivalent of the paired sample *t*-test used for continuous variables. The chi-square test was employed to analyse categorical data. When the minimum expected value was 5, Fisher's exact test was used. All the results are presented as two-tailed values with statistical significance if *p* values are =0.05. To adjust for all the other variables, and to make predictions, multivariate analysis was performed considering pneumonia occurrence (logistic regression) as dependent variables and age, gender, body habitus, comorbidities, ASA score, tumour localization, tumour stage, operative time, pain score at 24 and 48 hours after surgery, and type of surgery as independent variables.

## 3. Results

Demographics and disease-related data for each cohort are shown in Tables 1 and 2. There was no significant difference in terms of age, sex, weight habitus, ASA score, smoking habitus, chronic obstructive pulmonary disease (COPD), hypertension, and diabetes. Tumour stage and localization were similar, too.

**Figure 1:**
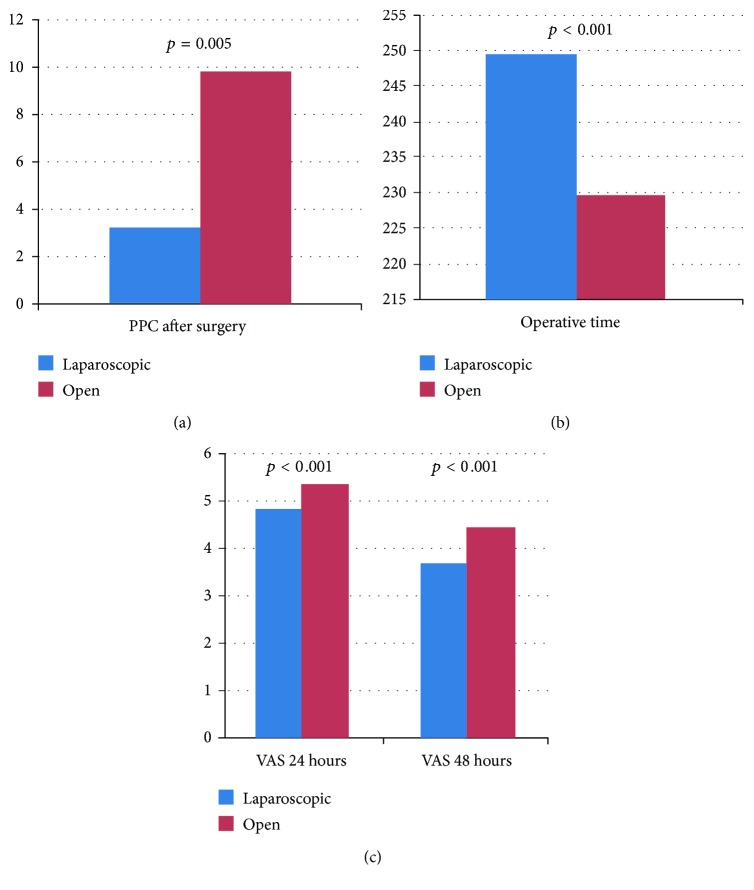
(a) Postoperative pulmonary complications after laparoscopic surgery and open surgery. (b) Operative time for laparoscopic surgery and open surgery. (c) Pain score after open surgery and laparoscopic surgery.

Overall, postoperative pulmonary complications (PPC) were significantly more common after open surgery (8 out of 242 patients (3.3%) versus 23 out of 235 patients (9.8%); *p* = 0.005) (Figure 1(a)).

Furthermore, laparoscopic surgery was associated with the increasing of operative time (249.6 ± 66.1 versus 229.6 ± 63.6; *p* = 0.001) (Figure 1(b)) and the reduction of pain at 24 hours (4.85 ± 1.58 versus 5.38 ± 1.52; *p* < 0.001) and 48 hours (3.74 ± 1.67 versus 4.46 ± 1.51; *p* < 0.001) after surgery (Figure 1(c)).

**Figure 2:**
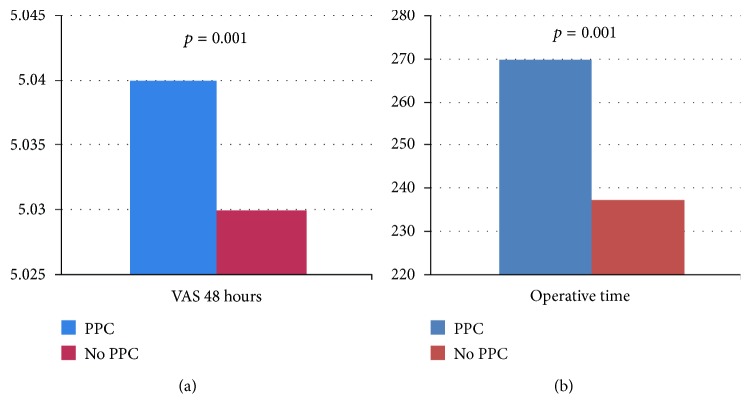
(a) Pain score in the PPC versus no PPC group. (b) Operative time in the PPC versus no PPC group.

Of interest, in a separate analysis comparing groups of patients with or without PPC occurrence after surgery, no association has been found for any patient or tumour characteristics, as shown in Table 2. In this analysis, PPC occurrence was associated with the increasing of pain after surgery (5.04 ± 1.62 versus 5.03 ± 1.58; *p* = 0.001) (Figure 2(a)) and with the increasing of operative time (270.06 ± 51.49 versus 237.37 ± 65.97; *p* = 0.001) (Figure 2(b)).

In fact, analysing separately the PPC occurrence in the group of patients undergoing laparoscopic versus open surgery, we found that PPC was associated with the increasing of operative time in the group of patients undergoing open surgery, but not in the group of patients undergoing laparoscopic surgery. In details, while among the laparoscopic group no difference was found (229 ± 63.8 minutes versus 247.7 ± 56.1 minutes; *p* = 0.41), among the open group, operative time was higher in the patients with pulmonary complications (246.5 ± 67.1 minutes versus 277.8 ± 48.5 minutes; *p* = 0.03).

Multivariate analysis (Table 3) confirmed that after adjusting for major patients and tumour characteristics, PPC occurrence was associated with open surgery (OR 1.37; 95% CI 1.009–1.052; *p* = 0.002), pain at 48 hours (OR 1.939; 95% CI 1.250–3.008; *p* = 0.003), and operative time (OR 1.009; 95% CI 1.001–1.018; *p* = 0.003).

## 4. Discussion

Colorectal cancer is the second cause of cancer-related deaths in western countries, and about one third of its tumours involve the rectum.

The outcome of surgery for rectal cancer has improved substantially during the past two decades because of the introduction of total mesorectal excision (TME) [14].

Although the introduction of TME in the early 1990s coincided with the progressive use of laparoscopic surgery in patients with colorectal disease, there is an ongoing debate on the optimal surgical resection of rectal cancers [9–12].

The most relevant clinical trials [9–12], focused on the surgical and oncologic outcomes, cannot draw a definitive conclusion, since the results are still controversial, but they provide the rationale to adopt laparoscopic surgery for rectal cancer.

Nevertheless, little is known about the relevance of nonsurgical complications after laparoscopic surgery and, in particular, with respect to postoperative pulmonary complications (PPCs). A deeper knowledge of this entity is mandatory since PPCs are associated with an increase in morbidity, mortality rates, length of stay, and healthcare costs and independently reduced the patient's median postoperative survival by 87% [15, 16].

The phenomenon is even more important in elderly patients, since they could be medically fragile, with high comorbidities and a reduced cardiopulmonary capacity. Concerns have been raised partly as a result of the respiratory and haemodynamic effect of pneumoperitoneum, the length of the procedures, and the extreme positions needed for exposure and dissection that are more pronounced in laparoscopic rectal surgery [17].

A systematic review of randomized controlled trials [5] on both colon and rectal cancers stated that although morbidity was poorly defined, for laparoscopic colectomies, a trend towards less pulmonary complications was observed.

Of interest, they found that only 22% of trials reported a mean or median age of included patients over 70 years. Subsequently, it is unclear if the results were representative for all patients.

The present series is the first comparative study specifically addressing the occurrence of postoperative pulmonary complications following laparoscopic and open rectal surgery in elderly patients.

Recently, some studies [18, 19] evaluated the short-term results of laparoscopic surgery for rectal cancer in elderly patients, obtaining controversial results. However, since they, once again, focused on surgical outcomes, the question on how laparoscopic surgery could influence the occurrence of postoperative pulmonary complications remains.

We found that postoperative pulmonary complications were significantly more common after open surgery. A multivariate analysis showed that after adjusting for major patients and tumour characteristics, only open surgery, VAS score at 24 hours, and operative time were associated with a slightly higher PPC occurrence. Reasons for increased PPC after open surgery could be numerous. As a first consideration, it is likely that the increased postoperative pain following an open procedure, compared to the pain perceived after laparoscopic surgery, can play a key role in the development of PPC. The fact that laparoscopy is associated with a reduced postoperative pain has been demonstrated by many studies [20–22]. A pathological domino effect is sometimes started by the larger incision needed in open procedures that can eventually lead to the development of a PPC. For an example, the patient's compliance to incentive spirometry is decreased by severe pain.

Furthermore, the intense postoperative pain often leads to the use of narcotics to be controlled, reducing the respiratory function (by suppressing the respiratory drive) causing the reduction of pulmonary hygiene and an increased number of atelectatic areas.

Also, postoperative ileus and extended hospital stay are PPC risk factors associated with open surgery, as proven by many studies [20–23]. It is worth mentioning that patients with postoperative ileus have a high risk of aspiration of the gastric content. Moreover, especially when the ileus is severe, a nasogastric tube is applied which represents a possible source of infection leading to PPC.

Finally, longer hospital stays are commonly associated with increased risk of nosocomial infections, particularly hospital-acquired pneumonia. Accordingly, we found that PPCs are associated with the increasing of pain after surgery. As a consequence, PPC occurrence is lower after laparoscopic surgery, with lesser pain in this patient population.

In addition, recent research suggests that prolonged operative duration can be associated with increased postoperative morbidity and mortality, especially postoperative pulmonary complications [24–31].

We found that PPC occurrence was associated with the increasing of operative time. However, even if operative time is higher for laparoscopic surgery, according to previous literature [32], the laparoscopic approach overcomes the association of operative time and PPC after surgery. Of interest, analysing separately the PPC occurrence in the group of patients undergoing laparoscopic or open surgery, we found that PPC was associated with the increasing of operative time in the group of patients undergoing open surgery, but not in the group of patients undergoing laparoscopic surgery, thus providing the rationale to hypothesize that laparoscopy could overcome the association between PPC occurrence and the increasing of operative time.

Some limitations of this study have to be addressed. The major limitation lies in its study design. Being a retrospective evaluation of a prospectively maintained database, there is the lack of patient randomization. However, multivariate analyses including all patients' characteristics have been performed to adjust for any result for all the other variables. Furthermore, although surgical approach was dependent on the clinical advice of each individual surgeon, a propensity score has been performed to exclude any related bias. In details, an attempt was made to check whether patients were matched [33, 34], based on the probability (propensity) of undergoing open or laparoscopic surgery. The predicted probability of undergoing one of the two procedures was estimated for each patient by using a multivariate logistic regression model in which the surgical procedure was the dependent variable and baseline patient and tumour characteristics (gender, age, weight habit, smoke, COPD, ASA score, hypertension, diabetes, tumour stage, and localization) were the independent variables. Thus, our results are encouraging to consider the laparoscopic surgery a safety and effective way to treat rectal cancer in elderly patients emphasizing that laparoscopic surgery reduces the occurrence of postoperative pulmonary complications. This study clearly provides the rationale for a randomized clinical trial, which would be useful to give a definitive conclusion.

## Figures and Tables

**Table 1:** Demographics and disease-related data in open versus laparoscopic group.

	Open	Laparoscopic	*p* value
*Age*	77.15 ± 6.17	78.02 ± 5.44	0.100
*Gender*			0.196
Male	143	109	
Female	92	133	
*ASA*			0.205
1	1	2	
2	98	123	
3	118	103	
4	18	14	
*Weight habit*			0.209
Normal	119	139	
Overweight	86	82	
Obesity	30	21	
*Smoke*			0.109
No	158	179	
Yes	77	63	
*COPD*			0.564
No	206	217	
Yes	29	25	
*Hypertension*			0.145
No	165	154	
Yes	70	88	
*Diabetes*			0.111
No	185	175	
Yes	50	67	
*T stage*			0.156
T0	8	14	
T1	13	16	
T2	54	48	
T3	138	153	
T4	22	11	
*Tumor localization*			0.147
Upper	37	46	
Middle	95	111	
Low	103	85	
*Neoadjuvant* (*pts*)	98	80	0.254
*Type of surgery*			0.512
Abdominoperineal resection	27	16	
Rectal resection	208	226	

**Table 2:** Demographics and disease-related data in PPC versus no PPC group.

	No PPC	PPC	*p* value
*Age*	77.15 ± 6.17	78.02 ± 5.44	0.100
*Gender*			0.573
Male	260	16	
Female	186	15	
*ASA*			0.522
1	3	0	
2	207	14	
3	208	13	
4	28	4	
*Weight habit*			0.468
Normal	244	14	
Overweight	156	12	
Obesity	46	5	
*Smoke*			1.000
No	315	22	
Yes	131	9	
			
*COPD*			0.070
No	399	24	
Yes	47	7	
*Hypertension*			0.324
No	301	18	
Yes	145	13	
*Diabetes*			0.831
No	337	23	
Yes	109	8	
*T stage*			0.192
T0	22	0	
T1	28	1	
T2	96	6	
T3	272	19	
T4	28	5	
*Tumor localization*			0.605
Upper	78	5	
Middle	190	16	
Low	178	10	
*Neoadjuvant*	157	12	0.331
*Type of surgery*			0.485
Abdominoperineal resection	41	1	
Rectal resection	186	22	

**Table 3:** Multivariate analysis of PPC occurrence after adjusting for major patients and tumour characteristics.

Variable	OR	95% CI	*p* value
Gender	1.069	0.359–3.183	0.905
Open surgery	1.037	1.009–1.052	0.002
VAS 48 hours	1.939	1.250–3.008	0.003
Operative time	1.009	1.001–1.018	0.003
Age	1.039	0.944–1.145	0.433
ASA score	1.555	0.651–3.716	0.321
Weight habit	1.367	0.653–2.866	0.407
Smoke	1.336	0.416–4.291	0.627
COPD	1.694	0.422–6.808	0.458
Diabetes	0.822	0.219–3.086	0.772
Hypertension	1.134	0.352–3.650	0.833
T stage	1.700	0.835–3.462	0.143
Tumour localization	0.731	0.328–1.631	0.444
